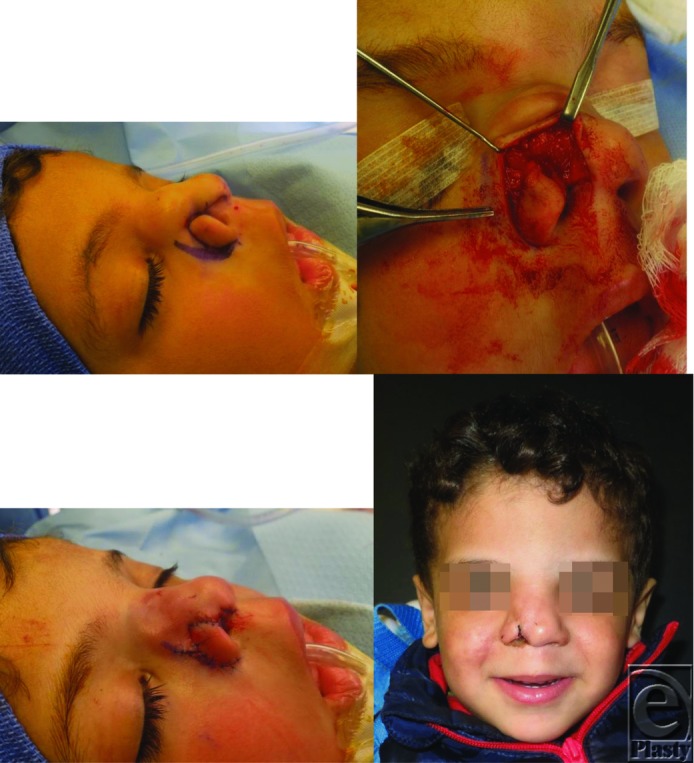# Tessier 1-13 Atypical Craniofacial Cleft

**Published:** 2015-06-17

**Authors:** Francesco Gargano, Karen Szymanski, Mitchell Bosman, Silvio Podda

**Affiliations:** ^a^Division of Plastic Surgery, St. Joseph's Medical Center, Paterson, NJ; ^b^New York Medical College, Valhalla

**Keywords:** atypical craniofacial cleft, craniofacial surgery, Tessier's classification, facial skeleton abnormalities, facial skeleton growth

**Figure:**
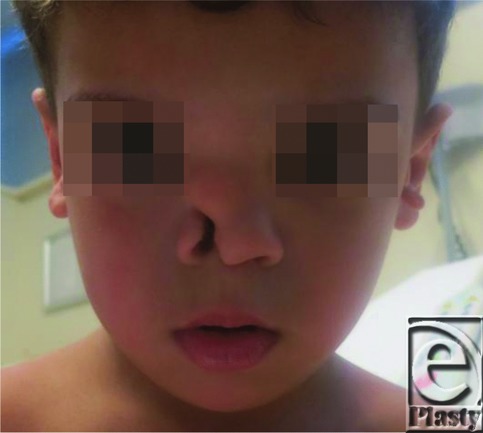


## DESCRIPTION

A 2-year-old boy presented with an atypical craniofacial cleft going through the right nostril with a soft-tissue extension of 2 × 1 cm. A computed tomographic scan showed bony involvement of the right nasal bone, right frontal bone, anterior cribriform plate anomalies, and no encephalocele.

## QUESTIONS

1. **What is the incidence and pathogenesis of the Tessier 1-13 cleft?**
2. **What is the classification of atypical craniofacial clefts?**
3. **What are the distinctive features of Tessier 1-13 cleft?**
4. **What is an appropriate treatment strategy regarding approach, timing, and sequence of reconstruction?**

## DISCUSSION

*Incidence and pathogenesis*. Tessier 1-13 cleft is an atypical craniofacial cleft with an extremely low incidence.^1^ The cleft results from failure of the nasal processes to fuse during weeks 5 to 8 of intrauterine life.^1^ Two major theories have been described as to the possible causes of facial clefting: the failure of fusion and the mesodermal penetration theories. Midface craniofacial development is heavily reliant on neural crest cell migration and differentiation. The differentiation of the neural crest cells is dependent upon many signaling protein pathways including the WNT and hedgehog pathways. Disruption of these pathways can lead to atypical craniofacial clefts.^2^

*Tessier's classification* is a clinical descriptive system of clefts rather than a system based on the involved structures or embryologic origin. The clefts are numbered from 0 to 14 in a counterclockwise fashion around the orbit. The value of Tessier's classification is the fact that it encourages the physician to look for malformations along the entire cleft direction.^3^ Each cleft path, the combination of 2 clefts, adds up to a value of 14.

*The distinct features of the type 1-13 cleft* are the involvement of the cupid bow, the alar dome notch at the middle third of the nostril rim, and the extension into the nasal dorsum passing medial to the normal canthal structures. The medial portion of the eyebrow can be dystopic without an obvious crease. Bony involvement includes the alveolus between the central and lateral incisors, the piriform aperture lateral to the anterior nasal spine, through the nasal bone at the junction of the nasal process of the maxilla, ethmoid labyrinth, and the olfactory groove of the cribriform plate, which is widened.^4^

*A treatment strategy* for each case is based on the potential soft tissues and bony growth and social interaction of the child. The initial intervention is typically limited to soft-tissue reconstruction, which can stabilize the bony discontinuity and possibly mold the underlying distorted facial skeleton. This may allow for a better social integration at school age. The bony reconstruction should be based on the normative residual growth within the facial skeleton, which requires correction. For mid-upper face, the earliest age for the procedure should be 5 or 6 years.

*Summary*. In our specific case, a significant soft-tissues disruption of 2 × 1 cm with superiorly displaced right ala was present. A complex design of 2 triangular 2 × 2-cm mucosal flaps was used to restore nasal lining. Nasal dorsal and right ala rotation flaps were used to reconstruct the overlying cutaneous defect. No extensive septum dissection or cartilage grafts were performed to avoid restriction of the growth potential.

**Figure:**